# *QuickStats*: Number of Deaths Resulting from Unintentional Carbon Monoxide Poisoning,* by Month and Year — National Vital Statistics System, United States, 2010–2015

**DOI:** 10.15585/mmwr.mm6608a9

**Published:** 2017-03-03

**Figure:**
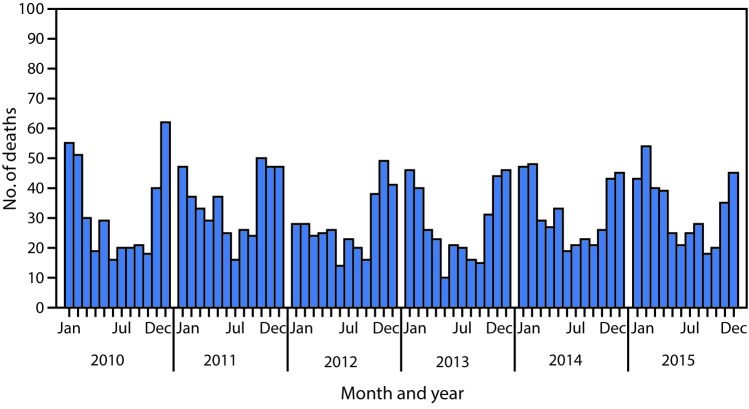
During 2010–2015, a total of 2,244 deaths resulted from unintentional carbon monoxide poisoning, with the highest numbers of deaths each year occurring in winter months. In 2015, a total of 393 deaths resulting from unintentional carbon monoxide poisoning occurred, with 36% of the deaths occurring in December, January, or February.

**Source:** National Vital Statistics System. Mortality public use data files, 2016. https://www.cdc.gov/nchs/data_access/vitalstatsonline.htm.

